# Pathogenic Mechanisms and *In Vitro* Diagnosis of AERD

**DOI:** 10.1155/2012/789232

**Published:** 2012-05-10

**Authors:** Dirk Schäfer, Steffen Maune

**Affiliations:** ^1^Allergie- und Intoleranzlabor, Medizinisch Klinik III, Friedrich-Alexander-Universität Erlangen-Nürnberg, Glückstraße 4a, 91054 Erlangen, Germany; ^2^Klinik für HNO-Heilkunde, Kopf- und Halschirurgie, Krankenhaus Holweide, Neufelder Straße 32, 51067 Köln, Germany

## Abstract

Aspirin-exacerbated respiratory disease (AERD) refers to chronic rhinosinusitis, nasal polyposis, bronchoconstriction, and/or eosinophilic inflammation in asthmatics following the exposure to nonsteroidal anti-inflammatory drugs (NSAIDs). A key pathogenic mechanism associated with AERD is the imbalance of eicosanoid metabolism focusing on prostanoid and leukotriene pathways in airway mucosa as well as blood cells. Genetic and functional metabolic studies on vital and non-vital cells pointed to the variability and the crucial role of lipid mediators in disease susceptibility and their response to medication. Eicosanoids, exemplified by prostaglandin E_2_ (PGE_2_) and peptidoleukotrienes (pLT), are potential metabolic biomarkers contributing to the AERD phenotype. Also other mediators are implicated in the progress of AERD. Considering the various pathogenic mechanisms of AERD, a multitude of metabolic and genetic markers is suggested to be implicated and were introduced as potential biomarkers for *in vitro* diagnosis during the past decades. Deduced from an eicosanoid-related pathogenic mechanism, functional tests balancing PGE_2_ and pLT as well as other eicosanoids from preferentially vital leukocytes demonstrated their applicability for *in vitro* diagnosis of AERD.

## 1. Introduction

Diagnostic tests assist the physician in assuring an appropriate treatment of the symptoms and as also the disease from which a patient is suffering. *In vitro* diagnostic tests are widely used in the practice of modern medicine. Nonsteroidal anti-inflammatory drugs (NSAIDs) are amongst the most frequently used drugs for the treatment of a variety of symptoms and diseases. Therefore, it is unsurprising that adverse reactions to NSAIDs arise in some patients. 

The diagnosis of NSAID-triggered, or exacerbated symptoms and diseases, is usually based on medical history or provocative challenge testing [1–8]. In some cases the latter is precluded on ethical grounds (e.g., pregnancy, children of young age), anatomical alterations (e.g., massive nasal polyposis), missing compliance of the patient (e.g., asthmatic experiences and therefore fear of life threatening symptoms), unavailability of specific technical and/or medical equipment (e.g., measurement of respiratory function, appropriate emergency unit), or inadequately trained staff [7, 8].

Several approaches attempted to diagnose and confirm NSAID-triggered symptoms and related diseases by *in vitro* diagnostic tools during the last 110 years. Some of them were discarded, others are under investigation. *In vitro* tests, and the results derived when they are used, frequently play a vital role in the overall diagnostic process. To ensure that each reader has the same basic knowledge, we will describe some rudimentary background information on terminology, suggested pathomechanism, test theory and test performance before discussing the *in vitro* test for diagnosis of NSAID-triggered symptoms and underlying diseases in more detail.

To some extent there is a known discrepancy of medical history and clinical symptoms upon exposure to NSAIDs, that is, that the provocation test shows negative outcome, whereas patients' history documented positive reaction. This may require an additional (*in vitro*) diagnosis to support the physician's decision for an appropriate treatment of the patient. Unfortunately, any diagnostic procedure, clinically and *in vitro*, is hampered by one or more inherent as well as exogenous factors. While some of them are known, most remain unknown, leading to some uncertainty of the test outcome.

**Figure 1:**
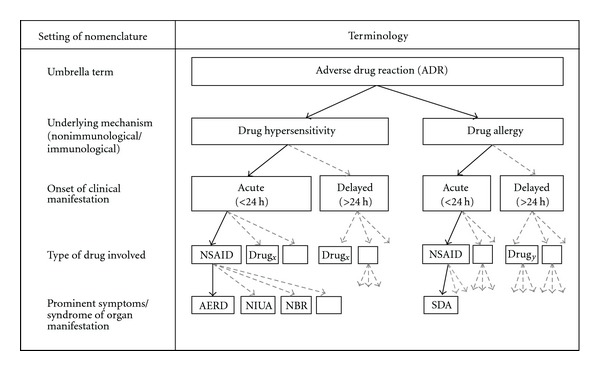
Allocation of terms used for adverse reactions to drugs. The diagram files the term AERD in the context of ADR, drug hypersensitivity, and drug allergy. The terms were gathered from “Report of the Nomenclature Review Committee of the World Allergy Organization” [7], and the proposed classification of allergic and pseudoallergic reactions to drugs that inhibit cyclooxygenase enzymes [9]; AERD: aspirin-exacerbated respiratory diseases, NSAID: nonsteroidal anti-inflammatory drugs, NIUA: NSAID-induced urticaria/angioedema, NBR: NSAID-blended reaction, SDA: single drug-induced anaphylaxis. Definition of ADR according to the World Health Organization [10]: any noxious, unintended, and undesired effect of a drug, which occurs at doses used in humans for prophylaxis, diagnosis, or therapy. This definition excludes therapeutic failures, intentional and accidental poisoning (i.e., overdose), and drug abuse.

The *nomenclature* for NSAID-triggered hypersensitivity reaction in medical literature might be confusing because of the diverse terms employed over last decades and the multiple clinical manifestations in humans. A list of terms used is given in Table 1, making no claim to be complete. Supporting the communication we consider the proposed terminology of “Report of the Nomenclature Review Committee of the World Allergy Organisation”, dating from 2003 [7]. This nomenclature is independent of the target organ or patient age group, but is based on the mechanisms that initiate and mediate reactions on our current knowledge, assuming that as knowledge about basic causes and mechanisms improves, the nomenclature will need further review. In this context “*hypersensitivity*” describes objectively reproducible symptoms or signs initiated by exposure to a defined stimulus at a dose tolerated by normal persons. The terminology “aspirin-exacerbated respiratory disease” (*AERD*) characterises physical reactions, underlying respiratory diseases, and inhibitors of cyclooxygenase (COX) and refers to the clinical syndrome of chronic rhinosinusitis (CRS), nasal polyposis, bronchoconstriction in asthmatics, and/or eosinophil inflammation in the upper and lower airways following the ingestion of NSAIDs blocking the COX-1 enzyme [9]. An assignment of AERD in the context of adverse drug reactions (ADR) and drug hypersensitivity is given in Figure 1.

*NSAIDs* are colloquially named “aspirin” or “aspirin-like drugs”. Aspirin, the trade name of acetylsalicylic acid (ASA), patented in 1899 by Bayer AG in Germany and in 1900 in the USA, was thereafter successfully marketed all over the world and still remains one of the world's safest, least expensive, and most frequently used drug [12]. *In vivo* absorption of salicylate and acetylsalicylic acid varies greatly from one individual to another but is reasonably constant within the same individual. Bound and unbound salicylate shows no differences in aspirin-tolerant and aspirin-intolerant patients, and the rate of deacetylation in serum is the same for aspirin-intolerant patients and normal controls [3, 13]. The pharmacological hallmark of acetylsalicylic acid and other NSAIDs is the blocking of COX-enzymes causing reduction and/or loss of prostaglandin (PG) production as demonstrated in 1971 by Ferreira and colleagues [14], Smith and Willis [15], and Vane [16]. Meanwhile there are several other NSAIDs known to inhibit the three known COX-isoenzymes, depending on their selectivity (an overview is given in Table 2, for review see [17]).

*The characterisation* of NSAID-triggered airway diseases, AERD, was first published by Widal et al. in 1922 [2] describing the symptoms, and was annotated by the eponym “*Syndrome de Widal.*” As it was written in French it was not until Samter and Beers popularised this syndrome 35 years later and the syndrome was annotated “*Samter's triad*” [3]. Severe cutaneous and systemic adverse reactions upon ingestion of “aspirin” was first documented in 1902 by Hirschberg [1], shortly after the market launch of aspirin. Nearly 90 years ago it was proposed that aspirin activates rather than inhibits peripheral chemoreceptors causing bronchoreactivity [18], increases blood flow, vascular permeability in skin and permeability of various membranes, bronchoconstriction, secretion of mucous glands, and alters in aspirin-intolerant patients [19]. Although NSAIDs, and aspirin in particular, are beneficial for their indicated use for most patients, these drugs account for 21–25% of all adverse drug reactions [20]. NSAIDs are well-known elicitors of upper and lower airway diseases and symptoms of other organs of adults as well as of children [5, 6, 8, 21].

*Symptom-based diagnosis of AERD* is usually performed by medical history, which is confirmed by *in vivo* provocation tests. For this purpose, oral, nasal, bronchial, or intravenous challenges with NSAIDs blocking the COX-1 enzyme are performed followed by measuring of nasal or pulmonary function [4–9, 12, 22]. The most common causes of adverse drug reactions are acetylsalicylic acid (~80%), ibuprofen (41%), and pyrazolones (~9%), but also nonselective COX-2 inhibitors are implicated. Medication, usage, and availability are most likely to be responsible for regional differences concerning published prevalence of adverse reactions to single NSAIDs. Therefore, the prevalence of aspirin hypersensitivity in the general population ranges from 0.6 to 2.5% and is up to ~30% in asthmatics also suffering from chronic nasal polyposis. The risk of severe adverse effects caused by challenge tests, ethical reasons, and/or other contraindications (see above) make an *in vitro* diagnostic test for AERD desirable [7, 8].

*In vitro diagnosis of AERD* is discussed in literature with some controversy, most likely based on insufficient and in part contradicting data of earlier and recent publications, as well as by former papers mentioning the unavailability of or inability to establish *in vitro* tests [4, 9]. Most clinicians have some acquaintance of their use. However, the underlying concepts pertaining to diagnostic tests in general, and to their use for diagnosis of a diseases in particular, are often less familiar, and perhaps less well understood. The current concepts point to the pathways of lipids (exemplified by eicosanoids) and other molecules related to them (e.g., cytokines, growth factors, cell surface markers, second messengers of cell signalling, enzymes and receptors). These will be summarised in brief and completed by some basic theoretical aspects.

*Eicosanoids* (notation introduced in 1980 by Corey et al. [23], a shorthand nomenclature of eicosanoids was given in 1987 by Smith and Willis [24]) are oxygenated metabolites of the (5*Z*, 8*Z*, 11*Z*, 14*Z*)-5,8,11,14-eicosatetraenoic acid, widely known as arachidonic acid (AA). Arachidonic acid is the main source of the eicosanoid cascade in humans involving more than 50 enzymes generating a multiplicity of eicosanoids [25, 26]. Concerning NSAID-triggered hypersensitivity and AERD, we selected and focused on the COX- and 5-lipoxygenase (5LO-) pathway. Both pathways are intimately linked to AERD and their implication is well documented (see subsequent literature). Beside these pathways and their metabolites, others such as those of cytokines, growth factors, or second messengers of signal transduction are also known to be implicated in AERD and related diseases. However, it is beyond the scope of this paper to cover all of them in known detail.

*Via the COX-pathway* prostanoids (i.e., prostaglandins (PG), thromboxane (TX)) are generated. The COX-pathway is blocked by NASIDs [14–16, 27] by acetylating the COX enzyme [28] and by causing inhibition of the conversion of arachidonic acid to PG [16]. COX-1 is constitutively expressed in most tissues and cells and is involved in cellular housekeeping functions. COX-2 is induced by inflammatory stimuli such as cytokines, growth factors, immunoglobulins, or bacterial toxins. Putative COX-3 mRNA is present in several tissues, including that from humans, but functional protein was still not found in humans. COX-3 is switched on later in inflammation and is suggested for biosynthesis of endogenous anti-inflammatory mediators. Its clinical relevance to COX-3 remains unproven. All COX isoenzymes are modified by NSAIDs with different efficacy (for review see [17, 27, 29]). The resulting metabolite PGH_2_ is further metabolised by PGE-synthase forming PGE_2_. The complexity of COX expression was demonstrated for human airways. There were no differences in the total number of cells stained for COX-1 and COX-2 irrespective of whether tolerant or intolerant to NSAIDs. The number and percentage of mast cells, however, that express COX-2 was significantly increased in patients intolerant to NSAIDs. Furthermore, the expression of COX-2 in epithelial and submucosal cellular was increased in asthmatics [30]. Additionally, the expression of COX-2 was downregulated in polypous tissue as well as in bronchial muscular cells from patients with AERD [31, 32]. PGE_2_ acts on at least four different seven-transmembrane-domain G-protein-coupled receptor subtypes, nominated EP_1_ to EP_4_. Binding on the EP_2_ or EP_4_ causes bronchodilatative effects, whereas binding to EP_1_/EP_3_ causes opposite effects [24].

**Figure 2:**
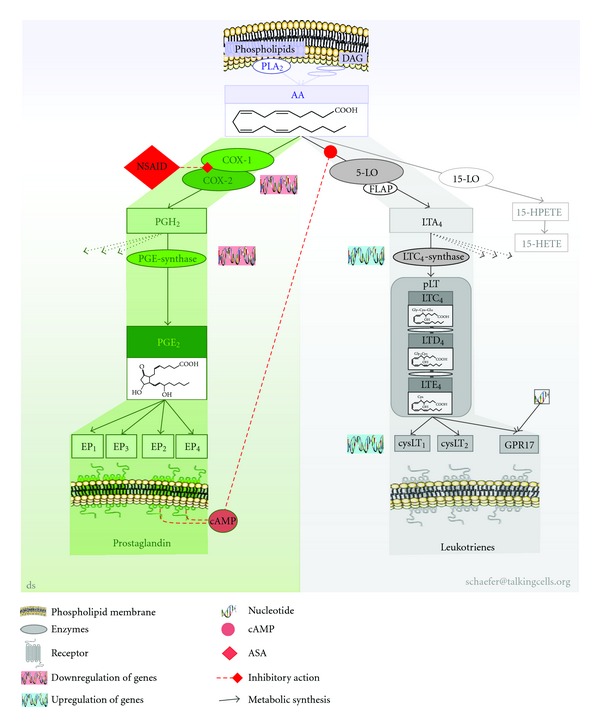
COX and 5-LO pathway in the metabolism of PGE_2_ and pLT for *in vitro* diagnosis of NSAID-triggered hypersensitivity. Simplified pictogram of eicosanoid pathways in the metabolism PGE_2_ and leukotrienes implicated for *in vitro* diagnosis of NSAID-triggered hypersensitivity. AA is enzymatically cleaved by calcium-dependent PLA_2_ from phospholipids (predominantly) or from DAG (minor amounts). AA is metabolised by the COX-pathway or 5-LO pathway (but also by several other pathways not figured out here). COXs generate PGH_2_, which is further processed by PGE-synthase forming PGE_2_ (other PGH_2_ metabolising pathways not mentioned here). PGE_2_ binds to EP subtypes of which EP_2_ and EP_4_ generate cAMP for signalling cascade. cAMP in turn causes negative feedback on the 5-LO pathway. AA is also metabolised by the 5-LO pathway (in part assisted by FLAP) generating LTA_4_. LTA_4_ is further processed by calcium-dependent LTA_4_-synthase forming amino acids bearing LTC_4_, which is exported and extracellularly metabolised by enzymes forming LTD_4_ and LTE_4_, collectively named pLTs All three LTs bind to cysLTs or GPR17 with differential selectivity. 5-LO: 5-lipoxygenase, AA: arachidonic acid, ASA: acetylsalicylic acid, cAMP: cyclic-adenosine monophosphate; cysLT: receptor of pLT, DAG: diacylglycerole, COX: cyclooxygenase, EP: PGE-receptor,GPR17: orphan receptor, binding pLT and nucleotides, HPETE: hydroxyperoxy-eicosatetraenoic acid, HETE: hydroxy-eicosatetraenoic acid, NSAID: nonsteroidal anti-inflammatory drugs, PLA_2_: phospholipase A_2_, PG: prostaglandin, pLT: peptidoleukotrienes.

*The lipoxygenase pathway* comprises several enzymes, generating several leukotrienes (LT). Focusing on the 5LO-pathway, LTA_4_ is generated from AA, which is further metabolised by the LTC_4_-synthase forming LTC_4_, containing three amino acid groups, which is actively exported in the extracellular space. An overexpression of the promotor of the LTC_4_-synthase gene was observed in some patients with AERD [33]. The amino acids are degraded by subsequent enzymatic processes forming LTD_4_ and LTE_4_. These metabolites have been named in 1960 by Brocklehurst as slow-reacting substances of anaphylaxis (SRS-A) [34] and were identified in 1982 by Hammarstrom and Samuelsson introducing the term leukotrienes for their occurrence in leukocytes and the characteristic chemical structure of conserved three conjugated double bonds (see Figure 2). These LT are characterised by a short half-life compared to other lipid mediators and are collectively named peptidoleukotrienes (pLT) based on their integral part of amino acids [35, 36].

The discovery of the 5-LO pathway caused an enormous interest in this area, largely displacing the “classic” prostaglandins. pLT are potent vaso- and bronchoconstrictors and have several other biological activities, including an ability to increase vascular permeability or to produce negative ionotropic effects in cardiac contractions [37, 38]. The pLT unfold their potential by currently three known seven-transmembrane-domain G-protein-coupled receptor types, named cysLT_1_ and cysLT_2_. A third dual orphan receptor GPR17 binds uracil nucleotides and pLT [39, 40]. Increased expression of cysLT_1_ and cysLT_2_ receptors is correlated to AERD [41–43].

The chemotactic metabolite LTB_4_, also generated from LTA_4_ but formed by a separate enzymatic pathway, is 100-fold less potent concerning bronchoconstriction and acts on a separate LTB_4_ receptor [38, 44]. Other lipid mediators are lipoxins (LX). LXA_4_ is known to inhibit LTC_4_ response and is decreased in patients with AERD [45, 46]. Further pathogenetic aspects in AERD are extensively reviewed by Palikhe et al. in this journal [47]. 

Attempting to condense the findings outlined above, a complex eicosanoid-protein interaction network has been discovered over the past decades, comprising lipid-derived mediators, second messengers, cytokines, receptors, enzymes, and activation of genes. Eicosanoids have a crucial role as mediators in inflammatory diseases like AERD. The enzymes and receptors of the eicosanoid cascade are found to be quite ubiquitous but also feature differences regarding distribution and expression in tissue and cells in normal circumstances as well as in patients with AERD. The COX-pathway can be attributed to the control of proliferative states, the 5LO-pathway to wound healing and tissue repair. Both pathways are embedded in other metabolic pathways, for example, the network of cytokines and neuropeptides, which in turn are also interconnected [48]. Gene expression and variability differs between AERD and NSAID-tolerant individuals with peculiarities with respect to ethnic background.

Some of these elements may directly interact with intracellular effectors to trigger multiple signalling cascades, while others act extracellularly. These components control and modulate cell migration, growth, proliferation, and activity of tissues and organs, which will result in differentiated reactions, unveiling symptoms like CRS, nasal polyposis, or asthma. A schematic overview is pictured in Figure 2.

## 2. Concept of Pathogenic Mechanisms

We will mention some of the known pathogenic mechanisms, elaborated in respect to AERD and to their supposed relevance to AERD, but limited to *in vitro* diagnosis of AERD and NSAID-triggered hypersensitivity.

Since the first description of adverse reactions to aspirin in airways [2], it is common knowledge that AERD is triggered by NSAIDs [3–9, 11–13, 21, 22, 30, 43, 46, 47, 49–52]. NSAIDS are known to modify the metabolism of unsaturated lipids, pinpointing eicosanoids [16]. Eicosanoids comprises a complex network of lipids essentially involved in the pathomechanisms of NSAID-triggered hypersensitivity or AERD.

NSAID hypersensitivity is characterised by an imbalance of eicosanoid synthesis (i.e., PGE_2_ and pLT) prior to as well as after exposure to aspirin. This was initially documented in 1999 as a result of analysing cultured peripheral blood cells [49] as well as nasal mucosa of the same patients [50]. The concept of the imbalance of eicosanoid synthesis [49] was taken up and approved recently by a theoretical study [51] and supported by former studies [52]. The genetic as well as functional modifications may be reasonable [33, 41–43, 46–51] but details are not fully understood, as expression of COX-2 is enhanced in macrophages [48] but no differences of COX-1 or COX-2 expression in patients with AERD and NSAID-tolerant individuals is found [30]. 

The reduced levels of PGE_2_ in AERD might be one initial factor for a diminished endogenous inhibition of the housekeeping function of PGE_2_, when activating the EP_2_ or EP_4_ receptor. These receptor types initiate the production of cyclic adenosine monophosphate (cAMP), a second messenger, after binding of PGE_2_ [48]. The synthesis of pLT is reduced by a cAMP-dependent intracellular signal transduction mechanism [11, 48, 52].

The reduced basal synthesis of housekeeping and induced PGE_2_ [11, 49–52], as well as the postulated [49] and validated overexpression of LTC_4_-synthase [33] and cysLT receptors [41–43] give rational arguments to explain at least in part the shift toward an elevated basal synthesis of pLT. This PGE_2_-pLT shift will be further elevated upon exposure to NSAIDs, but also by other agents initiating the eicosanoid cascade (e.g., cytokines like interleukine-1, or bacterial antigens). Thus, the reduced housekeeping/induced PGE_2_ most likely accounts for reduced production of cAMP, which is induced upon coupling of PGE_2_ on EP_2_ or EP_4_ receptors, but can be induced by other signal transduction pathways [48].

Thus, the diminished availability of the housekeeping (basal) and induced PGE_2_ will cause a reduced generation of suppressive acting endogenous cAMP upon exposure to COX-inhibiting agents.

In this context, PGE_2_, pLT, NSAIDs, cAMP, and other factors (e.g., bacterial toxins, availability of arachidonic acid, cytokines, and others) will most likely contribute in a highly complex manner to the multifactorial exacerbation of NSAID-triggered symptoms and diseases.

## 3. Theoretical Consideration of *In Vitro* Diagnosis

Since the latter half of the 1980s enzyme immunoassay (EIA) tests are widely used to screen and diagnose a multitude of diseases. Results are mostly classified by a binary outcome as “positive” (“reactive”) or negative (“nonreactive”), based on the protocols provided by the test manufacture and evaluation in the laboratory. The classification is the result of an ordered sequence of several steps, which had been initiated via the testing procedure.

Measurement repeatability and reproducibility are investigated during the approval process. For convenience, we will assume that the laboratory performing the test will maintain the complex process of the measurement system, and that the distribution of the results of “disease-free” and “diseased” individuals are normally distributed (see Figure 4).

In an “ideal” world these two normal distributions will not overlap. Regrettably the world of diagnostic testing is rarely unequivocally ordered. Many (currently and probably in perpetuity) unknown factors alter these distributions causing overlap to some extent. Regardless of where the test outcome threshold is situated on the measurement scale, some disease-free individuals and diseased (i.e., AERD) will be incorrectly classified as “negative” (known as “false-negative,” dark shaded area left-hand side of Figure 4) or “positive” (known as “false-positive,” grey area right-hand side of Figure 4), respectively. This represents one type of diagnostic test error.

Because any diagnostic test procedure has a single outcome threshold, moving the threshold to the right will reduce the false-positive results of disease-free individuals, but automatically will increase the false-negative error rate of the diseased individuals. Similarly, adjusting the threshold to the left will reduce the false-negative error rate, but automatically increases the false-positive error rate (i.e., classifying disease-free individuals as patients with AERD).

Only changing the distribution of test results in one or both groups would simultaneously reduce the rates of both types of diagnostic test errors (i.e., false-positive and false-negative results). Unfortunately, in realty this will not be practicable, due to the complex pathomechanisms underlying AERD, and the composition of the groups investigated like age, sex, medication, mentioned symptoms, interindividual variability of symptoms and syndromes, and our limited knowledge and understanding of the “plus-minus” clearly defined disease [53].

The terms* sensitivity (SE), specificity (SE), *and posttest probabilities in this concern refer to probability of an (*in vitro*) diagnostic test outcome, not to the equality of reagent or chemicals. Tests with a high sensitivity will correctly identify virtually all patients with NSAID-triggered hypersensitivity with a high probability; tests with high specificity identify all disease-free individuals correctly with a high probability. This becomes obvious when referring to Figure 4: sensitivity and specificity correspond to the area under the probability curve (i.e., the distribution) of patients with NSAID-triggered hypersensitivity (sensitivity of the test, on the right) and disease-free individuals (specificity of the test, on the left). Unfortunately, inadequacies in the pathological and clinical symptoms or comorbid components and symptom stage, including age and sex distribution of disease-free individuals as well as patients with disease were described ~30 years ago [54] and continued to hamper any diagnostic test [53].

What physicians are really interested in knowing is the extent to which a positive or negative test result accurately predicts the true status of the patient, that is, disease-free or patient with, for example, AERD. This is commonly referred to as the posttest probability of a disease (e.g., AERD), or predictive value of a positive test result (PPV). In case of a negative test result the posttest probability of being disease-free, that is, the predictive value of a negative test result (NPV) is of interest. These values depend on not only the sensitivity and specificity, but also on the pretest probability (or prevalence) of the disease (e.g., AERD). The mathematical algorithm connecting the three probabilities sensitivity, specificity, and prevalence is known as Bayes' theorem (originally published 1763 by R. Price [55] after the death of the English clergyman Thomas Bayes). It might be easier to grasp the sense of this relationship more directly than looking on the mathematical algorithm: the prevalence of AERD is arguable in respect to the supposed prevalence of 1.2 to 2.8% of a population [5, 8, 20]. However, as outlined before, there is some uncertainty concerning the *prevalence* (i.e., the pretest probability) of AERD due to the impossibility of diagnosing this syndrome by an absolute unfailing method. This marks a further limitation for “precisely” defining the outcome of an *in vitro* test by mathematical characteristics.

The probability term *likelihood ratio*, introduced in 1968 by Lustedt and popularised in the 1980s by Sacket et al. is a ratio of the two probabilities sensitivity and 1-specificity, describing the relative probability of a positive diagnostic test result in diseased individuals compared to disease-free individuals which can be calculated [56, 57]. For *ruling-in a disease* the likelihood ratio should be at least 1, preferably much higher (graphically this represents the area on the right site of the test threshold of Figure 4). In case of *ruling out a disease*, the likelihood ratio of a negative test result is chosen. These values should ideally be much smaller than one.

As easily deduced from the above-mentioned aspects, the definition of an optimal threshold is not only a question of statistics but rather depends on how the test result will be used. For screening purpose the threshold will be relatively low, resulting in higher false-positive outcomes. This requires additional diagnostic testing to ensure a therapeutic regime. In case of AERD a low threshold line will capture all patients, even those without obvious symptoms. The low threshold also covers the risk that a patient with a potential NSAID-triggered hypersensitivity but without obvious symptoms would undergo life-threatening reactions upon exposure to NSAIDs, would not be detected. Thus, the low threshold uncovers those patients with currently mild NSAID-triggered hypersensitivity for appropriate treatment before the disease worsens in the future. The latter is visualised, in part, by Figure 5, sketching schematically the course of NSAID-triggered hypersensitivity: The symptoms and underlying disease(s) do not relate in a uniform fashion, rather a pattern of exacerbation and remission is more like an exponentially growing sinus line. This pattern will be superimposed on the residual changes of the underlying disease and is a further challenge of *in vivo* and *in vitro* diagnosis of AERD.

## 4. *In Vitro* Diagnosis of AERD

The change in knowledge and concepts concerning the pathogenic mechanisms of AERD reflects the diversity of *in vitro* diagnostic approaches developed during the last century.

### 4.1. Serum-Specific IgE against NSAIDs (SIgNT)

The SIgNT examines serum or plasma collected from patients suffering from AERD and other manifestations of NSAID-triggered symptoms. The samples are filled into tubes coated with NSAIDs, including derivatives, or with NSAIDs/derivatives coupled to a carrier. After an incubation and washing step an anti-IgE or anti-IgG antibody labelled with a tracer (e.g., fluorochrome or chromogen finally converted by an enzyme) is added. Resulting values of the measurement will identify diseased patients if the value exceeds a predefined threshold (cutoff).

Underlying this approach was the observation, that adverse reactions to NASIDs displayed symptoms such as allergic reactions (the term “allergy” was introduced in1906 by von Pique as immunoglobulin mediated type of reaction [58]). Therefore, an immunologic reaction was assumed. Numerous attempts at detecting an antibody directed against Aspirin, derivatives thereof (e.g., anti-aspiryl antibodies), or to any other supposed NSAIDs failed to demonstrate an unequivocal antibody [3]. Even though antibodies were detected in 1940 by Butler et al. [59] and Zhu and colleagues [60], or propyphenazone-specific antibodies by the group of Ferreira [59], or were suspected by the group of Settipane [61]. These results have not been confirmed in the following decades [62, 63]. Also serum level of IgE in aspirin-intolerant patients did not differ from non-atopic population [61].

Nevertheless, these investigations contributed some substantial insights to our current understanding of AERD and to other NSAID-triggered symptoms as nonimmunologically mediated diseases. Thus, a SIgNT for the detection of antibodies directed to any NSAID could not be established and is not available for *in vitro* diagnosis of AERD.

### 4.2. Histamine Release Test (HRT)

The HRT examines urine samples from patients exposed to NSAIDs or supernatants of cell culture medium of peripheral blood cells (PBLs) incubated *in vitro* with varying concentrations of different NSAIDs.

The first approach (analysing urinary samples) would not be classified as an *in vitro* test as it affords an *in vivo* provocation/exposure of the patient. There are some essential drawbacks, arguing why this procedure (*in vivo* challenge) might not be suitable in some cases (because of, for example, ethical reasons, age, compliance, technical; see Section 1). Using PBLs for measurement of histamine release has to be designated as an *in vitro* diagnostic test.

The known bronchoconstrictive effect of histamine stimulated the attempt to look for an altered histamine release in patients with AERD [64]. Early investigations demonstrated elevated urinary excretion of a histamine metabolite [65] and elevated plasma histamine levels [66]. These measurements were, however, not confirmed in nasal lavage upon provocation [67, 68]. Preincubation of leucocytes with Aspirin failed to alter spontaneous or calcium ionophore-induced histamine release in patients with AERD [69, 70]. This was confirmed for bronchial lavage [71] and for leucocytes by our study performed *in vivo* as well as *in vitro* exposure [49, 50]. There are also some inconsistent results in former studies. Okuda and colleagues reported elevated histamine release induced by platelet-activating factor from leukocytes of patients with AERD [72], Hosemann and colleagues measured lower histamine content in polypous tissue of patients with AERD than in analgesic-tolerant patients [73], and the group of Stevensson reported elevated plasma histamine levels in only three of seventeen patients [74]. The low efficiency of histamine release by *in vitro* stimulation according to the CAST-protocol (see CAST) was also affirmed by a more recent study [75].

Even though the HRT was promising, as it depicts a pathomechanistic element of AERD, and it might be suitable to confirm AERD/NSAID sensitivity in specifically selected patients (e.g., with an underlying allergic comorbidity), it is not suggested for routine *in vitro* diagnosis of patients with AERD taking into consideration all data currently available.

### 4.3. Lymphocyte Transformation Test (LTT)

The LTT (synonyms are lymphocyte proliferation test or lymphocyte stimulation test) examines the activity of lymphocytes, notably of T-lymphocytes selected from PBLs upon exposure to varying NSAIDS at different concentrations. Most widely used for quantifying the proliferation is the measurement of ³H-thymidin uptake by dividing cells from samples of anticoagulated blood.

The relevance of the LTT as model system for analysing patients with hypersensitivity to Aspirin was discussed more than 40 decades ago [76–80]. Some NSAIDs do inhibit others from enhancing the proliferation, but this was not seen consistently [81–85].

A later study demonstrated an enhanced proliferation of normal lymphocytes, but a diminished ³H-thymidin uptake by lymphocytes from patients with AERD [86]. NSAIDs are considered suitable for LTT investigation [87]. But the inconsistency of results, and the more indirect relation of detecting lymphocyte proliferation to our current pathomechanistic understanding of AERD, often implicated unclear results. These findings questioned the clinical relevance of the LTT for the detection of adverse reaction to NSAIDs. Therefore, the LTT is actually not referred to be a suitable tool for *in vitro* diagnosis for patients with AERD.

### 4.4. Platelet Aggregation Testing (PAT)

The PAT examines survival and aggregation of platelets separated from venous PBLs. The platelets are exposed to varying concentrations of those NSAIDs which are of interest, for a defined time as validated by the performing laboratory.

Around 25 years ago it was suggested that platelets might have a pivotal role in AERD [88–93]. In a subsequent study, a group led by Picado detected no differences in any indices of platelet function studied between aspirin-tolerant and patients with AERD despite a slightly elevated aspirin-triggered PGF_2*α*_ release [94]. These results are somehow unexpected, as platelets are known to be potent producer of eicosanoids. Despite this approach and the implication of the platelet behaviour in NSAID-triggered symptoms, the PAT has not been approved for *in vitro* diagnosis of AERD.

### 4.5. Serum-PGF_2*α*_ Test (SPT)

The SPT examines serum selected from peripheral blood. Upon addition of a predefined concentration of ASA *in vitro*, samples are analysed using a radio-immunosorbent assay. Samples exceeding a predefined serum level of PGF_2*α*_ indicate patients with AERD.

This approach was introduced in 1991 by Willilams and colleagues and demonstrated no changes in PGE_2_ or PGD_2_ but lower plasma level of PGF_2*α*_ in patients with AERD before addition of aspirin, and elevated levels of PGF_2*α*_ after addition of aspirin, when compared to aspirin-tolerant asthmatics [95]. Small concentrations of aspirin given to platelet suspensions generated PGF_2*α*_ [96]. This confirmed the hypothesis of an NSIAD-triggered alteration of prostanoid metabolism and altered serum protein binding capacities in patients with AERD. Regrettably, there are no further publications documenting the routine use of this promising approach.

### 4.6. Mediators in Nasal Lavage (MNLT)

The MNLT examines nasal lavage collected from patients exposed *in vivo* to lysine aspirin. The nasal lavage is stored appropriately. After thawing and centrifugation the supernatant is analysed using specific enzyme immunoassays for two cytokines, MCP-3 and RANTES [97].

It was proposed that patients with AERD are characterised more likely by a chronic rather than an acute overproduction of MCP-3 and RANTES. The MNLT increased our pathomechanistic understanding of AERD, but an *in vivo* provocation step is presupposed. Hence, this approach does not meet the criteria of an *in vitro* test. Even though, the MNLT would be suitable to confirm AERD.

### 4.7. Exhaled Breath Condensate Eicosanoid Testing (EBCET)

The EBCET examines exhaled breath condensate of unexposed patients with AERD. The condensate is stored until analysis using an enzyme immunoassays specific for 8-isoprostanes, LTB_4_, and PGE_2_. Eicosanoid values exceeding a predefined threshold characterise patients with a positive test outcome.

This study was presented in 2002 by the group of Barnes, demonstrating elevated 8-isoprostanes and pLT (in ~50% of aspirin-intolerant asthmatics), no reduced PGE_2_ and unchanged LTB_4_ in exhaled breath condensate of patients with AERD exposed to NSAIDs [98]. These outcomes are in line with other studies also highlighting the implication of leukotrienes and prostanoids regarding diseases of the upper and lower airways [33, 49, 52, 99–103]. The EBCET was confirmed by the group of Szczeklik [104]. A very recent study extended the analysis of eicosanoids in exhaled breath condensate using gas chromatography/mass spectrometry and high-performance liquid chromatography/mass spectrometry. Before lysine aspirin challenge the amount of 5- and 15-HETE was higher in aspirin-intolerant asthmatics than in aspirin-tolerant asthmatics [105].

The approach of the EBCET depicts some of our recent knowledge on pathomechanisms concerning patients with AERD. This approach affords special equipment, mostly located in specialised centres. The EBCET, however, might become of some diagnostic value and would confirm AERD.

### 4.8. Cellular Allergen Stimulation Test (CAST)

The CAST examines cytokine-primed enriched basophilic granulocytes separated by density-gradient sedimentation from EDTA-anticoagulated venous PBLs. Cells are incubated for 15 up to 40 minutes with varying concentrations of variable NSAIDs in combination with complement factor 5a, or anti-IgE as positive control or vehicle. Reaction is stopped by freezing; supernatants are analysed by an enzyme immunoassay specified for cysteinyl-leukotrienes. Values from NSAID-stimulated samples have to exceed a predefined threshold (cutoff) of cysteinyl-leukotrienes (=pLT) released from a control sample to reveal a positive test outcome.

The CAST, introduced in 1993 by de Weck detects a biomarker with high relevance in AERD [106]. Different protocols were published. A sensitivity of 41 to 82% and a specificity of 82 to 100% were published. These variances are a consequence of method, as well as other details (e.g., sample preparation, selection of NSAID, duration of exposure, inclusion/exclusion criteria, age, sex, and number of patients/controls) [8, 73, 107–110]. Costimulation with complement factor 5a was claimed by the group of Weber to improve sensitivity [107]; they investigated patients with various underlying diseases. Low efficiency was reported with no diagnostic utility and superiority to the HRT [75]. Nevertheless, the CAST was successfully established for diagnosis of allergies [109].

According to a more recent study, the CAST uncovers a pathway which was different from the classical IgE-mediated pathway. CAST uses doses of ASA for *in vitro* stimulation causing nonspecific basophile activation, and thereby eliminates the usefulness of a cell based diagnostic test for AERD. Therefore, it was suggested that the CAST would have low value in diagnosing AERD and other diseases [108, 110].

### 4.9. Basophile Activation Test (BAT)

The BAT, also named FAST (Flow-cytometric Allergen Stimulation Test), examines basophilic granulocytes separated from EDTA-anticoagulated venous PBLs. Cells are incubated with varying concentrations of different NSAIDs for up to 40 minutes. Thereafter, basophilic granulocytes are double-marked with antibodies directed to IgE and CD63 (or CD203). The number of positively stained basophiles is measured using a fluorescence activated flow cytometer combined with appropriate software. A positive test outcome is defined by a laboratory-defined threshold (cutoff) of positively stained basophiles.

The BAT was introduced in 2000 by the group of de Weck [111]. CD63 is a cell surface glycoprotein that mediates signal transduction events that play a role in the regulation of cell development, (platelet) activation, growth and motility. CD203 represents a transmembrane ecto-nucleotide pyrophosphatase/phospho-diesterase-I enzyme (E-NPP), which cleaves phosphodiesters and phosphosulfate bonds. Both proteins are expressed on activated basophils. During the last decade follow-up studies were initiated to improve and ensure the technical procedures, thereby using the term BAT [112–115].

The BAT depicts an altered appearance of granulocytes, which are known to be implicated in AERD. Variable values of sensitivity (~10–64%) and specificity (~75–100%) were published depending on the protocols used (e.g., sample preparation, selection of NSAID, duration of exposure, inclusion/exclusion criteria, age, sex, and number of patients/controls). The clinical use of the BAT is controversially discussed [112–115], pointing to inherent factors influencing the opportunities and limitations of an *in vitro* diagnostic test.

### 4.10. Flow Cytometric Assay and CAST (Flow-CAST)

The Flow-CAST uses two techniques, the CAST (enzyme immunoassay) and BAT (flow cytometric assays). The outcomes of both tests are combined.

As reviewed in 2005 by the group of de Weck, the sensitivity and specificity varied depending on the NSAID tested [116]. The global sensitivity was annotated ~67%, the specificity 93%. Combination of BAT with CAST elevated sensitivity (to ~73%) but reduced specificity (to 71%). The Flow-CAST was proved for diagnosis of beta-lactam allergy [117]. It was proposed that in case of a negative result, a NSAID hypersensitivity cannot be excluded and a provocation challenge remains necessary if clinically indicated.

This approach demonstrates the usefulness of combining diagnostic procedures as mentioned in the introduction part, but demonstrates also the drawbacks as explained. From a practical point of view, performing both tests makes great demands on laboratory equipment as well as manpower, and therefore impacts on cost-effectiveness. The advantages of this procedure compared to others remain to be established.

### 4.11. Aspirin-Sensitive Patients Identification Test (ASPI Test)

The ASPITest examines PBLs exposed *in vitro* to varying concentrations of NSAIDs. The release of 15-hydroxyeicosatetraenoic acid (15-HETE) is analysed using an enzyme immunoassay specific for 15-HETE. Values exceeding a predefined amount threshold line (cutoff, ~6% exceeding basal release) identify patients with AERD [118].

The report by Kowalski and colleagues in 2005 concluded that the aspirin-triggered release of 15-HETE from PBLs does, to some extent, mimic the reactions observed *in vivo*. 15-HETE was detected in epithelial cells of nasal polypous tissue as well as in PBLs from patients with AERD, but not in asthmatics without NSAID hypersensitivity [31, 119, 120]. Already in 1991 the group of Picado demonstrated the *in vivo* evidence of elevated release of 15-HETE in nasal secretions of allergic patients [121]. It was demonstrated, that a PGE_1_ analogue (misoprostol) inhibited the aspirin-triggered 15-HETE release. A recent study investigating eight ASA-intolerant patients confirmed the elevated level of 15-HETE [120]. Variable values of sensitivity (~63–83%) and specificity (~50–82%) were published.

The ASPITest depicts a pathomechanistic link to AERD and obviously confirms the clinical finding in patients with AERD. Hitherto, there are only few promising publications and future studies will have to prove to which extent the ASPITest will be applicable for routine use for *in vitro* diagnosis of AERD and related diseases.

### 4.12. Functional Eicosanoid Testing and Typing (FET)

The FET examines PBLs of heparinised venous blood. PBLs are diluted in an appropriate buffer before exposure to ASA, neuropeptides, and arachidonic acid. The reaction is stopped by freezing. Upon thawing and centrifugation the samples are analysed using specific enzyme immunoassays for PGE_2_ and pLT. Measured data are calculated using appropriate software. The resulting individualised dynamic eicosanoid pattern is classified in values ranging from 0.0 to 3.0. This outcome is then more roughly classified as normal (0.0 to 0.5), mild (<0.5), moderate (<1.5), and severe (<2.5 to 3.0); these values also represent a probability of severity of the symptoms.

This approach was introduced in 1999 by Schäfer and colleagues and thereafter improved by integrating the growing knowledge of pathomechanistic concepts [11, 49–52, 122]. The FET depicts two biomarkers which are intimately involved in AERD and NSAID-triggered symptoms/diseases. First studies demonstrated the confirmation of clinically diagnosed AERD prior to, during provocation, and after successful treatment [123, 124]. Subsequent studies demonstrated the differentiation of non-airway-related but NSAID-triggered diseases [11, 125–127]. Others applied the FET for monitoring medical treatment in patients with AERD [128, 129] or characterisation of pathophysiological aspects [130]. Values for sensitivity and specificity varied depending on the underlying disease (airways: 96 and 89%, skin: 96 and 97%, gastrointestinal tract: 64–98 and 82–89%, resp.) [8].

The FET provides context-dependent cell-based confirmative as well as prospective information. This approach confirms AERD, but also differentiates and/or characterises underlying diseases of closely related symptoms; in addition, depending on the intended diagnostic challenge (as exemplified in Figures 6(a) and 6(b)). The FET differentiates obviously different symptoms of NSAID-triggered hypersensitivity of varying underlying disease. Future studies will have to demonstrate whether the FET, in addition to confirming or differentiating AERD, might provide some prognostic value in NSAID-triggered diseases.

## 5. Conclusions

During the last decades our knowledge concerning the pathogenic mechanisms, the terminology of NSAID-triggered symptoms and NSAID-exacerbated diseases (e.g., AERD) and the technical possibilities have continuously improved. This facilitated the development of new approaches for *in vitro* diagnosis, starting from no *in vitro* tests available 110 years ago to twelve *in vitro* tests developed during the last decades. Some characteristics and suggestions for intended use of the *in vitro* tests discussed are summarised in Table 3.

Our understanding of AERD and NSAID hypersensitivity moved form an immunoglobulin-triggered pathomechanism, diagnosed in the serum, to a multiplexed highly interconnected (eicosanoid) imbalance based on pathogenic understanding, diagnosing parameters from cell cultures, for example, genes, enzymes, mediators (lipids, cytokines, pH, and others), receptors, and others. A multitude of parameters were suggested. Surface marker of basophiles and lipid mediators remained to be the most promising biomarkers. Dynamic multiparametric approaches were favoured as compared to static single parametric approaches. A schematically simplified pictogram of the COX- and 5-LO pathway referred to for *in vitro* diagnosis is given in Figure 2.

**Figure 3:**
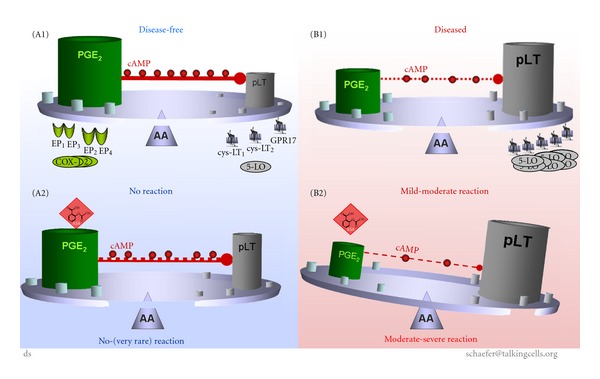
Causal concept of NSAID-triggered eicosanoid imbalance for *in vitro* diagnosis of AERD. The causal concept of NSAID-trigger eicosanoid imbalance for *in vitro* diagnosis of AERD is best allegorised as a tray balancing all parameters (which might be relevant for the pathway) on a needle. *Disease-free individuals*: housekeeping PGE_2_ balances synthesis of pLT (e.g., by induction of endogenous cAMP, which inhibits synthesis of pLT); expression of enzymes or receptors are unremarkable (A1). Upon exposure to NSAIDs the PGE_2_ level is diminished but remains high enough ensuring “uncritical” levels of pLT (even though cAMP might by diminished); expression of enzymes and/or receptors are not modified (A2). *Patients with AERD*: synthesis of housekeeping PGE_2_ is diminished, but still balances synthesis of pLT (e.g., by reduced endogenous cAMP); expression of enzymes (up regulation of LTC_4_-synthase) or receptors (up regulation of cysLT) can be mutated in some cases (B1). Exposure to NSAIDs/aspirin blocks the COX-pathway causing reduced synthesis of PGE_2_ (and consequently further reduced cAMP level), and consequently the metabolism of arachidonic acid is shifted to the 5LO-pathway provoking elevated synthesis of pLT; expression of enzymes and/or receptors may by altered, but not modified by NSAIDs (B2).

**Figure 4:**
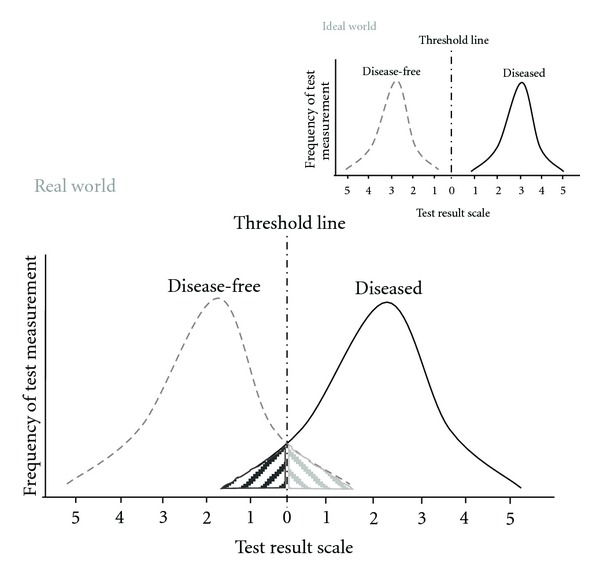
Framework for diagnostic test outcomes. Schema of “real” world diagnostic test outcomes; test measurement: clinical parameters like age, sex, ethnic group, height, weight, and so forth or analytical parameters like temperature, IgE, histamine, interleukins, lipid mediators; shaded areas exemplify the false-positive (false-negative) measurement of disease-free (diseased) individuals, respectively; insert: pictured “ideal” world.

**Figure 5:**
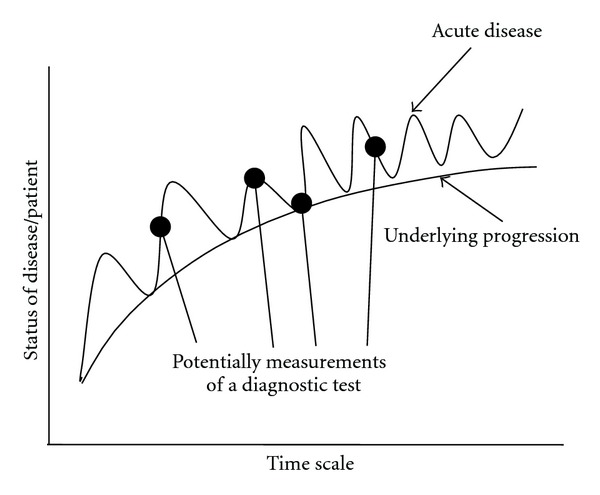
Hypothetical progress of AERD over time.

**Figure 6:**
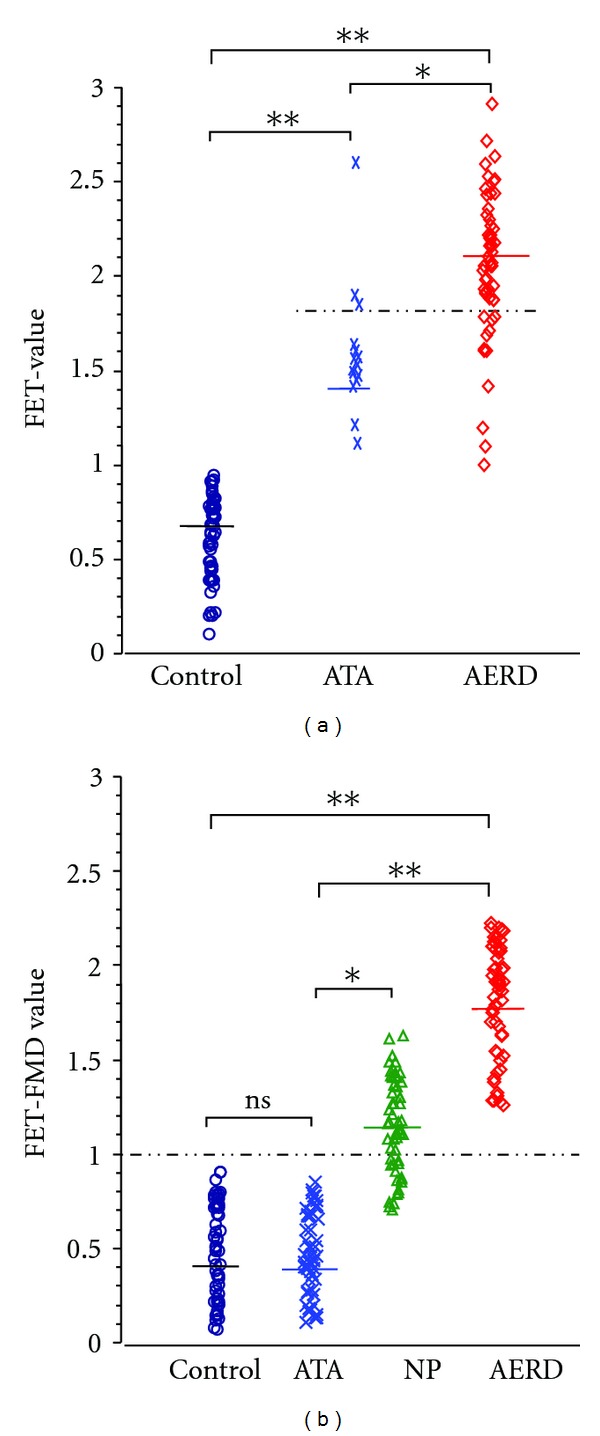
(a): FET and NSAID-triggered eicosanoid imbalance of individuals suffering from diseases with lower airway symptoms. The FET was performed and the FET values were calculated according to the total eicosanoid pattern score of [11] using PBLs. Patients suffering from NSAID-triggered bronchoconstrictive symptoms were confirmed and characterised by clinical and *in vitro* diagnosis. Allergy was ruled out by medical history, skin test, and *in vitro* test for total and specific immunoglobulin. The mean FET value (solid line) of controls, ATA, and AERD was 0.7, 1.4, and 2.1, respectively. FET values > 1.0 characterise patients with lower airway symptoms. FET values ≥ 1, 8 (dashed line, potential threshold) differentiate NSAID-tolerant asthmatics and patients with AERD; ATA: patients suffering from aspirin-tolerant asthma, AERD: patients suffering from aspirin exacerbated respiratory disease; (*n* = 53 for each group, *P < 0.05, **P < 0.01). (b): FET and functional metabolic differentiation of patients with and without NSAID-triggered eicosanoid of lower and upper airway symptoms. The functional metabolic differentiation (FMD) of subgroups of patient was achieved by *in vitro* provocation of PBLs and calculation of the FET value according to the total eicosanoid pattern score of [11], but by amending the FET value by subtracting the difference of the sum of the enzymatic capacity (EC) of PG- and LT-synthesis as well as the difference of the ASA- and neuropeptide-induced eicosanoid balances (EB) from the primary FET value (EC and EB were calculated according to [11]). The FET-FMD value takes into account two metabolites of the eicosanoid pathway and their *in vitro* modification by ASA and neuropeptide. The latter had been shown to be intimately implicated in hyperresponsiveness of airway ([11] and ref. therein). The FET-FMD value reveals the differentiation of ATA, NP, and AERD, but without discrimination of ATA and healthy controls. The mean value of FET-FMD (solid line) was 0.4, 0.4, 1.1, and 1.7, for controls, ATA, NP, and AERD, respectively. The threshold of FET-FMD was ≥1.0 (dashed line) for NSAID-triggered lower and upper symptoms of the airways. In conclusion, this approach confirmed and characterised NSAID-triggered symptoms by clinical and *in vitro* diagnosis. ATA: patients suffering from bronchial asthma, but tolerant to NSAIDs, NP: patients suffering from nasal polyposis, AERD: patients suffering from aspirin exacerbated respiratory disease with asthmatic symptoms; *n* = 53 for each group, ns: not significant, *P < 0.05, **P < 0.01. Allergy was ruled out by medical history, skin test and *in vitro* test of total an specific immunoglobulin.

The complexity of interacting parameters accounts for the initial situation where NSAIDs (see Table 2) start to act. If there is an imbalance of several metabolic and/or genetic parameters, the block of the COX pathway by NSAIDs will cause an exacerbation of one or more of prestage(s) of symptoms of a disease. Diagnosing the balance and imbalance of the eicosanoid cascade might be fundamental for diagnosing and treating NSAID-triggered diseases (see Figures 1 and 3). These approaches might be hampered by high individual variability of underlying diseases, genetics, enzymatic/cellular function/activity, and by inclusion and exclusion criteria during sample collection for *in vitro* diagnosis. The (*in vitro*) test outcome has to be carefully interpreted by an appropriately trained physician and researcher concerning terminology, inclusion, and exclusion criteria, test theory, and last but not least, the most recent hypothesis and models of pathogenic mechanisms.

All *in vitro* tests, currently available, consider our current pathogenic and clinical understanding of AERD. But the intended use by the clinician or researcher will also account for the selection of the most appropriate *in vitro* diagnostic procedure (e.g., screening purpose, confirmation of a clinical diagnosis, individual risk assessment, proof of, prognostic probability, and/or differentiation of symptomatic appearance, monitoring of treatment, effect of single drugs, and many more). Considering the limitations of clinical diagnosis of AERD (see above), the “provocation” test is yet designated as “gold standard” in clinical diagnosis, but is usually restricted to confirm acute physical reactions of hyper reactive lower airways and requires the necessity for patients' provocation. But this “gold standard” will fail if AERD is still not thoroughly distinctive, a prognostic goal has to be considered, or provocation is precluded.

The relevance of the diagnostic test outcome and its interpretation will improve if the users of an *in vitro* diagnostic procedure consider all information provided. In this concern, functional cellular *in vitro* approaches mimic some of the complex *in vivo* processes seen in patients with AERD. The imbalance of eicosanoids might be a rational decision-making model for *in vitro* diagnosis of AERD as well as NSAID-triggered hypersensitivity. Future research will demonstrate whether and which functional *in vitro* approach will prove to be the “gold standard” of *in vitro* diagnosis of AERD to support treatment of patients with AERD and related diseases.

## Figures and Tables

**Table 1:** Terms used for reactions of NSAID-triggered hypersensitivity. NSAID: nonsteroidal anti-inflammatory drugs; COX: cyclooxygenase.

Terms used	Predominant manifestation/location of symptoms	Supposed underlying pathomechanism
Syndrome de Widal	Airways	Pathomechanism unknown, hyperreactivity/-sensitivity to aspirin and aspirin-like drugs
Samter's triad	Airways	Pathomechanism suspected to altered sensitivity of chemoreceptor, hyperreactivity of airway mucosa to aspirin and aspirin-like drugs
Aspirin idiosyncrasy	Anywhere, ubiquitous	“Peculiarity” of hypersensitive reaction to aspirin and aspirin-like drugs which is not elicit by immunoglobulin-mediated/immunologic reactions, but by dysfunction or loss of function of enzymes
Aspirin allergy	Anywhere, Ubiquitous	Involvement of immunoglobulin-mediated/immunological reactions directed to aspirin and aspirin-like drugs
Pseudoallergic reaction to aspirin	Anywhere, ubiquitous	Reaction to aspirin and aspirin-like drugs, causing symptoms as seen by allergic, reactions (i.e., immunoglobulin-mediated/immunologic), but without involvement of immunological reactions
Aspirin intolerance	Anywhere, ubiquitous	Pathomechanism unknown/not defined, but aspirin and aspirin-like drugs are not tolerated by an individual
Aspirin sensitivity	Anywhere, ubiquitous	Pathomechanism unknown, but hyperreactivity/-sensitivity to aspirin and aspirin-like drugs, symptomatic description
Aspirin-sensitive asthma	Lower airways	Hyper-reactivity to aspirin and aspirin-like drugs causing airway obstruction
Aspirin-induced asthma	Lower airways	Pathomechanism unknown, but initiated/induced by aspirin and aspirin-like drugs
Aspirin-exacerbated respiratory disease (AERD)	Airways, systemic	Exacerbated by NSAIDs blocking COX-1 pathway
NSAID-induced rhinitis and asthma (NIRA)	Airways	Exacerbated by NSAIDs blocking COX-1 pathway
NSAID-induced urticaria/angioedema (NIUA)	Skin, systemic	Exacerbated by NSAIDs blocking COX-1 pathway
Single drug-induced urticaria/angioedema (SDUA)	Skin	Exacerbated by a single NSAID blocking COX-1 pathway
Multi-drug-induced urticaria/angioedema (MDUA)	Skin	Exacerbated by multiple NSAIDs blocking COX-1 pathway
Single drug-induced anaphylaxis (SDA)	Systemic	Sensitisation to a single NSAID blocking COX pathway, suggested immunoglobulin-mediated/immunologic pathomechanism
NSAID-blended reaction (NBR)	Airways, skin	Pathomechanism unknown; not AERD, not NIRA, presumably not immunoglobulin-mediated/immunologic

**Table 2:** NSAIDS: classification, mechanism of action, representative structures. NSAIDs can be classified based on their chemical structure or mechanism of action; older NSAIDs were classified by chemical structure or origin, newer ones more often by their mechanism of action; COX: cyclooxygenase, 5-LO: 5-lipoxygenase.

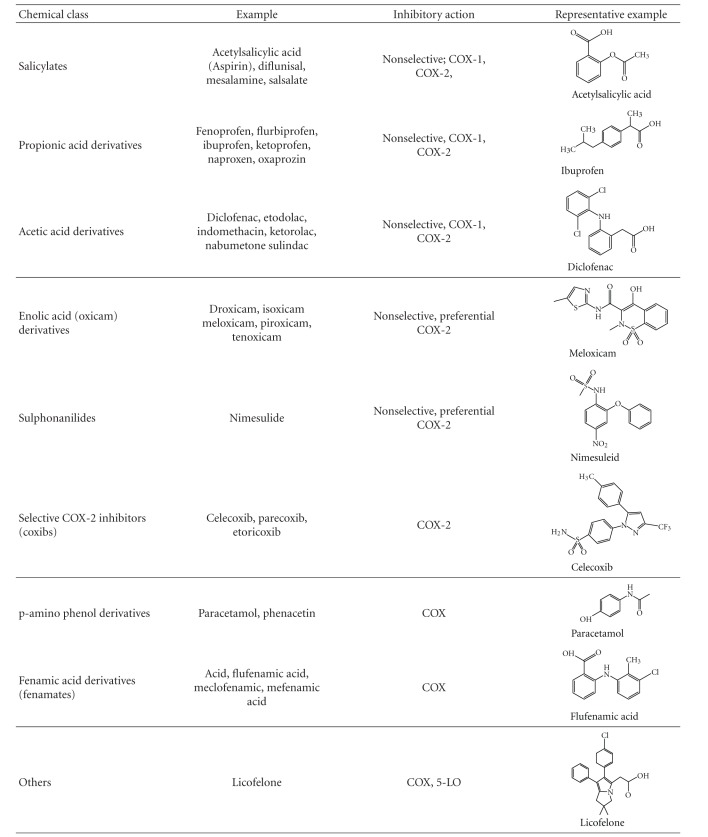

**Table 3:** Selected characteristics and suggestion for use of tests described *in vitro* diagnosis. ASPI Test: aspirin-sensitive patients identification test, BAT: basophile activation test, CAST: cellular antigen stimulation test, EBCET: exhaled breast condensate eicosanoid testing, Flow-CAST: flowcytometric assay and CAST, HRT: histamine release test, FET functional eicosanoid testing and typing, LTT: lymphocyte transformation test, MNLT: mediators of nasal lavage test, PAT: platelet aggregation test, SIgNT: serum-specific immunoglobulin E against NSAIDs test, SPT: serum-PGF_2*α*_ test; LT: leukotrienes, PG: prostaglandin, CD: cluster of differentiation, HETE: hydroy-eicosatetraenoic acid; SE: sensitivity, SP: specificity; PPV: positive predictive value, NPV: negative predictive value; n.v.d.: no values described — not suggested, (—) suggested, actually not in use, ? suggested upon further validation, (+) suggested with restrictions, + suggested.

*In vitro* test	Test parameter	Test sample	SE(%)	SP (%)	PPV (%)	NPV (%)	Suggestion for *in vitro* diagnosis
SIgNT	IgE, IgG	serum	n.v.d.	n.v.d.	n.v.d.	n.v.d.	—
HRT	histamine	culture medium, PBLs	n.v.d.	n.v.d.	n.v.d.	n.v.d.	—
LTT	proliferation	lymphocytes	n.v.d.	n.v.d.	n.v.d.	n.v.d.	—
PAT	aggregation	platelets	n.v.d.	n.v.d.	n.v.d.	n.v.d.	(—)
SPT	PGF_2*α*_	serum	n.v.d.	n.v.d.	n.v.d.	n.v.d.	?
MNLT	MCP-3, RANTES	nasal lavage	n.v.d.	n.v.d.	n.v.d.	n.v.d.	—
EBCET	8-isoprostane	exhaled breast condensate	n.v.d.	n.v.d.	n.v.d.	n.v.d.	?
CAST	cysLT	culture medium, basophiles	41–82	82–100	~96	~78	(—)
BAT	CD63, CD203	culture medium, basophiles	60–70	<90	~95	~56	(—)
Flow-CAST	cysLT, CD63	basophiles	~10–67	~75–100	n.v.d.	n.v.d.	(+)
ASPI Test	15-HETE	culture medium, PBLs	63–83	>50–82	79	86	?
FET	PGE_2_, pLT	culture medium, PBLs	96 (64–98)	83 (82–89)	90 (70–96)	93 (69–98)	+
